# Assessment of infection prevention and control readiness for Ebola virus and other diseases outbreaks in a humanitarian crisis setting: a cross-sectional study of health facilities in six high-risk States of South Sudan

**DOI:** 10.11604/pamj.supp.2022.42.1.33906

**Published:** 2022-06-11

**Authors:** Alex Yao Sokemawu Freeman, John Pasquale Rumunu, Zacharia Afram Modi, Argata Guracha Guyo, Abraham Alberto Uyu Achier, Nyankiir Ajing Jefor Alor, Taban David Kilo Ochan, Walter Awatta Ochan, Sylvester Maleghemi, Kibebu Kinfu Berta, Olushayo Oluseun Olu

**Affiliations:** 1 World Health Organization, Juba, South Sudan,; 2 Ministry of Health, Juba, South Sudan

**Keywords:** Infection prevention and control, water, sanitation, hygiene, Ebola virus disease, disease outbreak, humanitarian crisis, South Sudan

## Abstract

**Introduction:**

the study was conducted to assess the readiness and capacity of the core components of infection prevention and control and water, sanitation and hygiene in health facilities to effectively contain potential outbreaks of Ebola virus and other diseases in South Sudan.

**Methods:**

it is a descriptive cross-sectional study which was conducted in health facilities in six high-risk States of the country from September 2020 to December 2021. Data was collected using a structured questionnaire and analyzed with Microsoft Excel software.

**Results:**

one hundred and fifty-one (151) health facilities with a total bed capacity of 3089 were enrolled into the study. Overall, the least prepared infection prevention and control, water and sanitation core components in ascending order were the coordination committee structure (13.19%), guidelines and SOPs (21.85%), vector control (22.02%), staff management (30.63%), and training received (33.64%). The best prepared components in descending order were integrated disease surveillance and response capacity (69.83%), medical waste management system (57.12%) and infrastructure compliance (54.69%).

**Conclusion:**

the findings of this study which is comparable to those of other studies in similar settings validates the perception that Infection Prevention and Control/Water, Sanitation, and Hygiene (IPC/WASH) capacity and readiness is inadequate in South Sudan. To scale up these core components, we recommend development and implementation of a comprehensive and long-term infection prevention and control strategic plan as part of the country’s broader health sector recovery planning.

## Introduction

Infection Prevention and Control (IPC) is aimed at preventing avoidable infection of patients and healthcare workers at health facility and community levels [1]. It is a key determinant of Universal Health Coverage (UHC) and health security [2]. It plays a critical role in preventing antimicrobial resistance in healthcare settings and in preparing health care systems to prevent and respond to current and future infectious disease threats [3]. Core components include standard precaution measures such as hand hygiene, the use of personal protective equipment (PPE) by designated health workers, early detection and isolation of cases of infectious diseases, effective medical waste management, and safe burial [4,5]. Available scientific evidence has identified poor IPC practices as a major driver of transmission of infectious diseases outbreaks, particularly viral hemorrhagic diseases [6]. Unfortunately, such outbreaks often occur in humanitarian settings like South Sudan where the health system is weak, and IPC infrastructure, supplies, staffing, and training are rudimentary [7].

Water, Sanitation, and Hygiene (WASH) plays an essential role in IPC, particularly in healthcare settings. Infection prevention and control and WASH are two sides of the same coin; thus, safe WASH contributes to enhanced IPC, patient, and health workers´ safety, and vice versa [8]. A robust WASH system significantly prevents and controls diseases such as schistosomiasis, trachoma, soil-transmitted helminths, and diarrhea diseases, including cholera [9]. In recent times, both concepts have gained much traction with the emergence and re-emergence of infectious diseases such as Severe Acute Respiratory Syndrome (SARS), COVID-19, Ebola Virus Disease (EVD) and other viral hemorrhagic fevers, which require strong IPC/WASH [10-12] .

South Sudan experiences recurrent incidents of emergencies regularly; a significant number of these emergencies are outbreaks of infectious diseases which often results in high morbidity and mortality. The country’s southern parts sit in the ecological zone of the EVD and yellow fever, which is contiguous with the same zone in neighboring Democratic Republic of Congo (DRC) and Uganda [13]. The country also continues to experience repeated outbreaks of other infectious diseases such as Rift Valley fever, measles, cholera, etc. [14-17]. At the onset of the 2018, EVD outbreak in Ituri and North Kivu Provinces of DRC, South Sudan and three other countries that border DRC (Burundi, Rwanda and Uganda) were designated as high-risk for cross border importation of the EVD, which necessitated heightened preparedness activities [18]. Thus, the country implemented various EVD preparedness interventions from August 2018 to December 2019 [19]. The COVID-19 outbreak in Wuhan, China, in December 2019 further dictated sustained preparedness and response interventions for infectious diseases in the country. The country recorded its first case of COVID-19 on April 5, 2020 and thus moved from the preparedness to response phase, further requiring a strong outbreak prevention and control system [20].

South Sudan scaled up IPC/WASH interventions due to the increasing threats of disease outbreaks, however, there is a paucity of reliable data on the status of IPC/WASH interventions in the country. This is a major constraint to evidence-based prioritization, planning, implementation and monitoring of IPC/WASH interventions. Thus, IPC interventions are often ad-hoc rather than proactive and systematic. Furthermore, the technical knowledge and capacities of national and international agencies involved in implementing IPC/WASH interventions in the country are often weak, affecting both the quantity and quality of interventions. Given the exposure of South Sudan to threats of cross border importation of EVD, recurrent outbreaks of infectious diseases and the ongoing COVID-19 outbreak, which require effective IPC/WASH interventions for prevention and control, the importance of having reliable data for evidence-based planning, implementation and monitoring of interventions cannot be overemphasized. An IPC/WASH study was thus conducted in selected health care facilities in the country.

The objective of the study were to assess the readiness and capacity of the core components of IPC/WASH in health facilities to effectively respond to and contain potential outbreaks of EVD and control the ongoing COVID-19 outbreak in the country and to establish baseline information for planning, implementation, supervision, monitoring, and evaluation of short- and long-term IPC/WASH interventions. The study also sought to raise awareness about and provide evidence for advocating for more investments in IPC/WASH in the country. In this research article, we present and discuss the key findings, conclusions, and recommendations of the study.

## Methods

**Study design and sampling method:** a descriptive cross-sectional study on the capacity and readiness of the core components of IPC/WASH to respond to infectious disease outbreaks was conducted in health facilities in six high-risk States of South Sudan from February 2020 to December 2021. The three Equatoria States were initially enrolled into the study in 2020. With the report of the first case of COVID-19 in the country on April 5, 2021 and subsequent declaration of an outbreak, three more States, namely, Western Bahr el Ghazal, Lakes and Jonglei, classified as high risk for both EVD and COVID-19 transmission, were enrolled into the study. A purposive sampling method was used to identify between 14 to 16 health facilities per State. Inclusion criteria were any operational health facility with permanent structures and at least two or more healthcare workers.

Study setting: South Sudan, a country experiencing a chronic humanitarian crisis, is bordered by Uganda in the South, DRC in the South West, Central Africa Republic in the West, Sudan in the North, Ethiopia in the East and Kenya in the Southeast. These borders are porous with free population movements between the neighboring countries for economic and sociocultural reasons. The country has a 2021 population of 11.4 million and landmass of 619,745 km2 [21]. It is divided into ten States and three administrative areas which are grouped into the three historical Provinces namely Bahr el Ghazal, Equatoria and Greater Upper Nile. Equatoria is in the southern part of the country and is sub-divided into three States namely Central, Eastern and Western Equatoria. Equatoria shares borders with Uganda and DRC; thus, it is classified as high-risk for cross border transmission of EVD outbreaks. Western Bahr el Ghazal, Lakes and Jonglei States share borders with Equatoria Province in their southern parts and are also classified as high-risk for EVD transmission. As of the time of this study, all six States had recorded cases of COVID-19 which further justified their inclusion in the study. Healthcare delivery in South Sudan is through a network of Primary Health Care Units (PHCUs), Primary Health Care Centres (PHCCs), County (General) and State referral hospitals at the formal level while informal health services are delivered through the Boma Health Initiative at the community level. Oversight for the healthcare system of the country is provided by the State and National Ministries of Health.

Data collection: quantitative data was collected using a structured questionnaire (Annex 1) adapted from the WHO guidelines on core components of IPC programmes at the national and acute health care facility levels [2] and Water and Sanitation for Health Facility Improvement Tool (WASH FIT) [9] both of which represent the minimum requirements for IPC/WASH in health facilities. The questionnaire contained 112 questions grouped into twelve sections that reflect the IPC core components and WASH in health facility minimum requirements (Table 1). Data was collected by six IPC/WASH experts from WHO and the Ministry of Health. Before the commencement of data collection, the data collectors were trained on the study objectives, methods, and the data collection tool. The questionnaire was pre-tested by the data collectors and revised accordingly before deployment and commencement of actual data collection. In each health facility, the data collectors introduced themselves and explained the study objectives and data collection mechanism to the head of the facility. The questionnaire was then administered to either the head of the health facility or the officer in charge of IPC/WASH. A physical assessment of the facility was conducted to verify the responses to the questionnaire. Global Positioning System (GPS) coordinate of each health facility was obtained and recorded on the questionnaire to facilitate mapping.

Data analyses: the data was cleaned, entered and analyzed with Microsoft Excel software. Twelve indicators identified based on expert consensus were analyzed (Table 1). The average score of the response for each indicator was calculated and tabulated by State. Based on the overall score achieved in the twelve indicators, each State was assigned to one of four IPC/WASH status levels: not available, basic, intermediate, and advanced (Table 1). A score of 50% and above is considered acceptable within the context of South Sudan. The frequency distribution of the assessed health facilities was calculated and tabulated.

**Table 1:** infection prevention and control/water, sanitation, and hygiene (IPC/WASH) core components, criteria for assessment and scorecard

Core component/Indicator assessed	Criteria for assessment	Scorecard
Hygiene and sanitation	Twenty-four (24) questions to assess the cleaning procedures, availability of latrines, handwashing, shower, washing and drainage facilities	0%: not available (the IPC/WASH core component has not been implemented)
Integrated Disease Surveillance and Response (IDSR) capacity	Seven (7) questions to measure capacity of health facility to conduct IDSR	25%: basic (some core components of IPC/WASH are available but not sufficient; further improvement is required)
Infrastructure compliance	Nine (9) questions assessing compliance of health facility infrastructure with IPC/WASH guidelines and Standard Operating Procedures (SOPs)	50%: intermediate (most core components of IPC/WASH are available and appropriately implemented. The facility should continue to improve the scope and quality of implementation and focus on the development of long-term plans to sustain and further promote the existing IPC/WASH core functions)
Medical waste management system	Twenty (20) questions assessing capacity for management of sharp, soft contaminated, organic, and hazardous waste	100%: advanced: (IPC/WASH core components are fully available and implemented according to the WHO recommendations and appropriate for the needs of the facility)
Staff management	Six (6) questions to assess availability, training and job description of IPC/WASH staffing in the health facility	
Type of training received	Seven (7) questions assess IPC/WASH training of healthcare workers	
Vector control	Four (4) questions to measure the implementation of key vector control interventions such as indoor residual spraying, use of bed nets and window screens for control of mosquitos	
Quantity of water supply	Ten (10) questions to assess the quantity and quality of water supply to the health facility	
IPC/WASH guidelines and SOPs	Three (3) questions to assess availability and use of IPC/WASH guidelines and SOPs	
IPC/WASH committee structure	Nine (9) questions to assess the availability, functionality, objectives, and funding of IPC/WASH committees	
IPC/WASH supplies	Eight (8) questions to assess the availability of IPC/WASH supplies such as soap, hand sanitizer, light personal protective equipment and post-exposure prevention kits	

Ethical clearance and approval: administrative clearance for this study was provided by the Ministry of Health of South Sudan. World Health Organization provided the ethical clearance (WHO e-Pub no: ePub-IP-00331783-EC).

## Results

**Figure 1:**
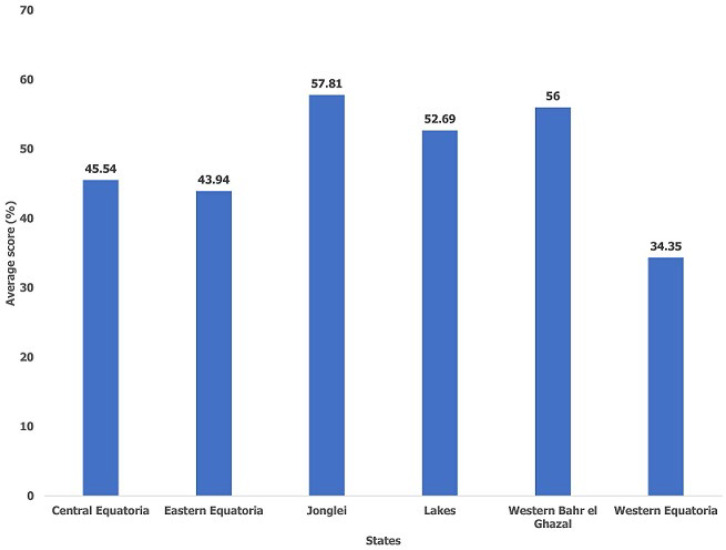
average score of infection prevention and control/water, sanitation, and hygiene (IPC/WASH) readiness in South Sudan by State - December 2021

**Figure 2:**
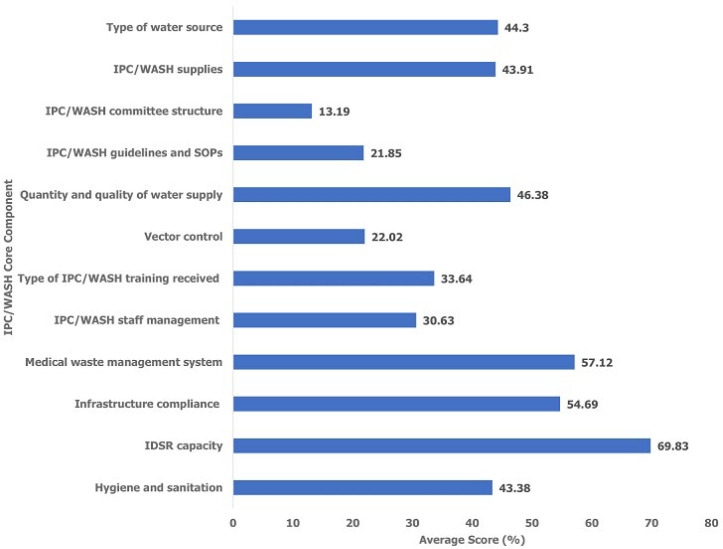
average score of infection prevention and control/water, sanitation, and hygiene (IPC/WASH) readiness by core component in South Sudan - December 2021

One hundred and fifty-one (151) health facilities with a total bed capacity of 3089 in six States were enrolled into the study. Almost half (49%) of the surveyed health facilities were PHCCs while the rest were PHCUs (25%) and hospitals (14.51%) (Table 2). Western Equatoria State (34.45%) was the least prepared State followed by Eastern Equatoria (43.94%) and Central Equatoria (45.54%) (Figure 1). Overall, the least prepared IPC/WASH core components in ascending order were the IPC/WASH committee structure (13.19%), IPC/WASH guidelines and SOPs (21.85%), vector control (22.02%), IPC/WASH staff management (30.63) and IPC/WASH training received (33.64%) (Figure 2). The best prepared components in descending order were IDSR capacity (69.83%), medical waste management system (57.12%) and infrastructure compliance (54.69%) (Figure 2). The IPC/WASH committee structures, guidelines and SOPs were generally poor across all the six States (Table 3). Western Equatoria State scored very low in IPC/WASH committee structures (8.39%), guidelines and SOPs (11.42%), IPC/WASH staff management (18.87%) and vector control (19.40%) (Table 3). Eastern Equatoria State scored very low in vector control (3.44%) and IPC/WASH committee structure (18.33%) while Lakes State scored very low in IPC/WASH training received. Jonglei State scored very low in IPC/WASH committee structure (4.17%) (Table 3).

**Table 2:** summary of health facilities assessed by State

State	Total no. of health facilities assessed	Total no. of beds	Type of facilities			
			PHCU	PHCC	Hospital	Others
Central Equatoria	48	1563	3	25	9	11
Eastern Equatoria	20	165	8	9	2	1
Jonglei	6	282	0	3	1	2
Lakes	7	321	0	5	1	1
Western Bahr el Ghazal	16	486	0	11	5	0
Western Equatoria	54	272	27	21	4	2
Total	151	3089	38	74	22	17

**Table 3:** average score of infection prevention and control/water, sanitation, and hygiene (IPC/WASH) readiness by core component and State of South Sudan - December 2021

IPC/WASH core component (%)	State					
	Central Equatoria	Eastern Equatoria	Jonglei	Lakes	Western Bahr el Ghazal	Western Equatoria
Hygiene and sanitation	48.64	40.83	64.93	53.87	52.89	30.71
IDSR capacity	51.50	76.96	95.24	77.55	90.40	70.17
Infrastructure compliance	66.8	52.92	62.5	57.54	58.16	41.72
Medical waste management system	56.84	52.31	88.54	66.07	82.13	46.06
IPC/WASH staff management	33.85	44.79	40.28	38.10	34.38	18.87
Type of IPC/WASH training received	40.40	50.54	21.43	1.53	17.44	31.70
Vector control	25.79	3.44	54.17	27.68	27.38	19.40
Quantity and quality of water supply	50.16	33	44.17	64.64	62.97	40.08
IPC/WASH guidelines and SOPs	20.83	33.37	16.67	51.19	34.38	11.42
IPC/WASH committee structure	14.06	18.33	4.17	19.44	21.01	8.39
IPC/WASH supplies	44.33	45.94	65.10	70.54	69.34	28.56
Type of water source	41.98	56	51.67	45	57.81	34.90

## Discussion

This study sought to establish the capacity and readiness of the core components of IPC/WASH in health facilities to prepare for and respond to disease outbreaks and enhance the safety of patients and health workers in South Sudan. To the best of our knowledge which is based on an exhaustive literature review, it is the first documented study that comprehensively assessed the status of IPC/WASH in health facilities in the country thus, its findings will form the baseline for planning, supervision, monitoring, and evaluation of IPC/WASH interventions moving forward. The findings showed a lack of readiness of most IPC/WASH components to prevent disease outbreaks except for IDSR capacity, infrastructure compliance and medical waste management system. There was a generally poor readiness of IPC/WASH committee structure, vector control and IPC/WASH supplies components across most of the study area. The study showed weak IPC readiness in the three Equatoria States which had the highest risk for EVD transmission.

The general lack of readiness of most IPC/WASH components that were observed in this study is similar to the findings of Lowe *et al*. in their study of IPC in eight conflict-affected countries [22] and those of Pathmanathan *et al*. and Fofanah *et al*. in Sierra Leone [23,24]. This trend may be attributed to several reasons. Due to several years of war, a chronic humanitarian crisis and neglect, the health system of South Sudan is chronically weak and barely able to support good quality healthcare services, including IPC/WASH. For instance, gross underfunding with less than 3% of the country´s Gross Domestic Product allocated to the health sector annually is inadequate to finance the delivery of good quality health care services [25]. Likewise, the dearth of healthcare workers, weak supply chain management system for essential medicines and medical supplies and inadequate service delivery also constrain delivery of essential IPC/WASH interventions. The civil conflicts of 2013/2014 and 2016 contributed to further decimation of an already weak health system thus resulting in an acute on chronic problem. Lack of access to several parts of the country for long periods due to insecurity, harsh terrain and natural disasters such as flooding often disrupts the delivery of essential medical supplies and healthcare services, including IPC/WASH. In addition, the concept of IPC/WASH in healthcare facilities is relatively new to the country which may account for limited knowledge and skills of healthcare managers and workers on organizing it. Furthermore, lack of a comprehensive national IPC/WASH strategic plan due to lack of evidence-based information, poor coordination and inadequate oversight of IPC/WASH intervention perhaps are the most critical challenges of IPC/WASH in the country, which is similar to the findings of Cooper *et al*. in Liberia [26].

The high scores observed in the IDSR capacity, infrastructure compliance and medical waste management system components may be associated with ongoing health interventions before and during the study period. As part of efforts to respond to the chronic humanitarian crisis in the country, humanitarian partners led by WHO made massive investments in an Early Warning Alert and Response System (EWARS) to address the need for good quality and real-time data for timely detection and response to epidemics. The EWARS built a foundation for improving disease surveillance which resulted in significant improvements in IDSR capacity all over the country [27]. Similarly, an ongoing EVD preparedness programme to the tune of USD 30.5 million at the time of the study resulted in investments in several components of IPC/WASH [28]. For instance, the four EVD isolation units built and major infrastructural improvements made in several healthcare facilities in the three Equatoria States could have accounted for the high score observed in the infrastructural compliance component. Although the medical waste management component had an acceptable score, we observed lack of materials and systems to enhance proper waste segregation at points of generation, inadequate numbers of standard high temperature incinerators and properly designated waste management areas for effective infectious waste treatment and final disposal in many of the health facilities. These are required for the management of hazardous medical wastes and are thus critical gaps which need to be addressed to improve IPC/WASH in the country moving forward. These findings are similar to those of Lowe *et al*. and Forrester *et al*. in their assessment of IPC in Liberia [22,29].

The IPC/WASH committee structure which requires minimum funding and efforts to implement was the weakest among all the IPC/WASH components which is corroborated by the findings of Tartari *et al*. which showed that low income countries were less likely to have functional IPC/WASH programmes [30]. This finding is perhaps due to the fact that that most of the health facilities in the country lack the required staffing strength in terms of quantity, cadre and quality to constitute the required IPC/WASH committees which is the same as the findings of Fofanah *et al*. in Sierra Leone [24]. Inadequate understanding of and lack of capacity for health coordination at the subnational level may have also constrained the establishment of these committees and their proper functioning. Furthermore, inadequate knowledge of international IPC/WASH norms, standards and guidelines may also contribute to the poor functionality of these committees.

The below average score observed in the vector control component across all the States points to gaps in the malaria control programme, which is primarily responsible for this component hence the need for strengthening the national malaria prevention and control programmes and ensuring greater coordination and collaboration between it and the the IPC/WASH programme at all levels. Although similar to the findings of another study conducted in Uganda [31], the below average score observed in the IPC/WASH supplies component in the three Equatoria States despite an ongoing EVD preparedness programme with a huge procurement component of IPC/WASH supplies is surprising. The same trend was also observed in the IPC/WASH guidelines and SOPs component across all the States except for Lakes. These findings may be attributed to poor coordination, duplication of efforts and inadequate access which were challenges which were earlier described by Olu *et al*. [19].

Limitations: the above findings should be interpreted within the context of three key limitations. Only six and seven health facilities were sampled in Jonglei and Lakes States due to access constraint. Given this small sample size, the findings in both States may not be a true reflection of the IPC/WASH readiness. The purposive sampling method used may have resulted in selection bias in which the more readily accessible and possibly the most functional and good performing health facilities were selected. Lastly, the involvement of some Ministry of Health IPC/WASH officials in the data collection process may have introduced interviewer bias. The data collectors may have focused on their predetermined perceptions which may affect their assessment of the various components of IPC/WASH. This bias was addressed by rigorous screening, selection, training and supervision of the data collectors and physical verification of the responses.

## Conclusion

The findings of this study which is comparable to those of other studies in similar settings validates the perception that IPC/WASH capacity and readiness is inadequate in South Sudan. This observation is confounded by the fact that the massive IPC/WASH investments made during the EVD preparedness programme of 2018 to 2020 and the ongoing COVID-19 response programme seem not to have yielded the anticipated outcomes. The inadequate capacity and readiness of the IPC/WASH programme observed in this study are mainly due to the chronically weak health system, lack of a comprehensive national IPC/WASH strategy and inadequate knowledge and funding of IPC/WASH interventions in the country. Other factors include weak capacity for IPC/WASH planning, implementation, supervision, monitoring and evaluation in the country. Furthermore, most of the international IPC/WASH norms and standards may not be realistic in view of the peculiar context of South Sudan.

Moving forward, we propose four main recommendations based on our findings. First, the results of this study should be used to develop and implement a long-term IPC/WASH strategic plan for the country as part of the broader health sector strategic, health system recovery and annual humanitarian response planning. This strategic plan should have a clear monitoring and evaluation framework based on the baseline established by this study and should be actualized through annual IPC/WASH operational plans. Second, the study results should be used to scale up advocacy and resources mobilization for IPC/WASH in the country. Third, international IPC/WASH norms, standards and guidelines should be adapted to suit the South Sudan context. For instance, it may be practically impossible to constitute IPC/WASH committees in every health facilities due to inadequate staffing hence the need to innovate. Fourth, establishment of national and State level platforms for IPC/WASH coordination is critical to coordinate and monitor progress.

### What is known about this topic

- Infection prevention and control/water, sanitation, and hygiene (IPC/WASH) is a critical component of outbreak preparedness, response, prevention and control in humanitarian settings such as South Sudan;
- There is inadequate evidence-based information for the planning, implementation, supervision, monitoring and evaluation of IPC/WASH interventions in the country;
- Despite the exposure of the country to threats of disease outbreaks, the readiness of the IPC/WASH programme to adequately respond to these outbreaks is unknown.

### What this study adds

- This study identifies the key gaps in the readiness and capacity of IPC/WASH programme in the country to prevent and control diseases outbreaks;
- The study further provides baseline information for future planning, supervision, monitoring and evaluation of IPC/WASH interventions at health facility level.
